# 

**Published:** 1907-05

**Authors:** 


### BOOKS RECEIVED.

The Nursling. The feeding and hygiene of premature and full-term infants. By Pierre Budin, Professor of Obstetrics, University of Paris; Director of the Clinique Tarnier. Authorised Translation by William J. Maloney, M. B., Ch. B., Fellow of the Obstetrical Society of Edinburgh, with an introduction by Sir Alexander R. Simpson, M.D., Emeritus Professor of Midwifery and Diseases of Women and Children, University of Edinburgh. Large 8 vo, pp. 223. With 111 diagrams in color and other illustrations. London: The Caxton Publishing Company. New York: Imperial Publishing Company. 1907. (Price, $6.00.)

---

International Clinics. A Quarterly of Illustrated Clinical Lectures and especially prepared articles on Treatment, Medicine, Surgery,

Neurology, Pediatrics, Obstetrics, Gynecology, Orthopedics, Pathology, Dermatology, Ophthalmology, Otology, Rhinology, Laryngology, Hygiene and other topics of interest to students and practitioners. Edited by W. T. Longcope, M.D. Volume 1, Seventeenth series. 1907. Philadelphia and London: J. B. Lippincott Co. (Cloth, $2.00).

---

A Manual of Obstetrics. By A. F. A. King, M.D., Professor of Obstetrics and Diseases of Women in the Medical Department of the George Washington University, Washington, D. C., and in the Medical Department of the University of Vermont, etc. Tenth edition, enlarged and thoroughly revised. 12mo., 688 pages, with 30 illustrations and three colored plates. Lea Brothers & Co., Philadelphia and New York, 1907. (Cloth, $2.75, net.)

---

The Technic of Modern Operations for Hernia. By Alexander Hugh Ferguson, M.B., M.D., Professor of Clinical Surgery, Medical Department of the University of Illinois; Professor of Surgery at the Chicago Post-Graduate Medical School. Royal octavo, pp. 336. Illustrated by reproductions of original drawings from the author's collection. Chicago: Cleveland Press. 1907. (Cloth $4.00; half morocco $5.00.)

---

Physical Diagnosis. With case examples of the Inductive Method. By Howard S. Anders, A.M., M.D., Professor of Physical Diagnosis in the Medico-Chirurgical College, Philadelphia. Octavo, pp. 475. With 88 illustrations in the text and 32 plates. New York and London: D. Appleton and Company. 1907. (Price, $3.00.)

---

Essentials of Chemistry and Toxicology for the Use of Students in Medicine. By R. A. Witthaus, A.M., M.D., Professor of Chemistry, Physics and Toxicology in Cornell University. Thirteenth edition. Revised by R. J. E. Scott, author of "The State Board Examination Series." New York: William Wood and Company. 1907. (Price, $1.00.)

---

Proceedings of the American Medical Editors' Association. Thirty-seventh annual meeting held in Boston, June 5, 1907. Joseph MacDonald Jr., M. D., Secretary.

